# Tuberculosis

**Published:** 1904-05-28

**Authors:** 


### Progress in Medicine and Surgery.

### TUBERCULOSIS.

**Early Diagnosis.**—The importance of early diagnosis is especially seen in the results of sanatorium treatment, the success of which largely depends on its early application. Clarke[1] emphasises the following points as indications of early phthisis :—1. Increased pulse-rate. 2. Gradual loss of weight without obvious cause. 3. Digestive disturbance. 4. Tubercle bacilli, if present in *considerable numbers* in the sputum. 5. "Vesiculo-bronchial" breathing, which consists of a short, roughened inspiratory and a prolonged, blowing expiratory sound. 6. Small crepitant râles at the end of expiration. Of less value are : 1. Fever, which may be absent. 2. The tuberculin test, which is unreliable. 3. The shape of the chest, which may be normal. The *x*-rays may give confirmatory evidence. Clarke attaches importance to family history, and still more to personal environment as indications of possible tuberculosis ; Lister[2] notes that in tuberculous families a greater risk attaches to the weakly, whose chest development and weight are below normal. Woods Hutchison,[3] however, has shown that contrary to the received opinion, the phthisical chest is round instead of flat. The normal ratio of transverse to antero-posterior diameter is about 100 : 70 in adults, whereas in 40 tuberculous cases in America it was 100 : 79·5, and in 42 cases at the Brompton Hospital 100 : 79·6. Colbech, at the Victoria Park Hospital, found the ratio in 75 cases 100 : 80·3, and from measurements on early cases considered that the shape of the chest was antecedent and not consequent to the pulmonary disease. The small râles which Clarke mentions are also to be detected by Cybulski's method. This consists of listening to the patient's respiration with the observer's ear about 2 to 4 inches from the patient's open mouth. Remouchamps[4] states that fine crepitations which appear to originate in the larynx, resemble the scratching of a fine nib on paper, and are usually most marked at the end of expiration, are pathognomonic. Although in other lung conditions adventitious sounds may be transmitted by the mouth, all differ from this "laryngeal crepitation ;" moreover, phthisis may even be excluded if this sign is absent after repeated examinations. Petruschky[5] considers that even before physical signs can be found in the lungs the bronchial glands are often diseased, which gives rise to tenderness on firm pressure along the spine between the second and seventh dorsal vertebræ inclusive. With regard to the tuberculin test Marmorek[6] considers that the mere presence of the tubercle bacillus without anatomical lesions is enough to give a positive reaction. If a limited number of bacilli are injected into guinea-pigs, they give the reaction in 15 minutes, but after a longer interval phagocytosis interferes with it. He attributes the febrile symptoms to the formation of a peculiar toxin by the bacilli under the direct influence of the tuberculin.

**Prophylaxis and Public Health.**—The exclusion of tuberculous immigrants apart from pauperism has provoked much opposition in medical circles in the United States. Knopf[7] and Robinson[8] both strongly condemn the new law, pointing out that though phthisis is communicable it is not infectious in the ordinary sense of the word. A new law enacted in the State of New York giving local authorities power to veto the construction of sanatoria in their areas is also vehemently opposed. Robinson advocates extension of sanatorium accommodation, but also insists on improved sanitation and ventilation in tenements and better instruction for the labouring classes in hygiene and cookery. Lawson[9] approves of the "Garden City" scheme, and of the passing of bye-laws against expectoration in public places. Ambler[10] emphasises the following prophylactic points : disinfection of sputum, prohibition of promiscuous coughing and spitting ; boiling of all eating and drinking utensils of consumptives ; prohibition of kissing and handling of food intended for others by consumptives ; and reduction of dust to a minimum in houses. He would not isolate cases, but would forbid their frequenting public halls and entertainments. He considers that young children run considerable risk of infecting themselves from dirt and dust of floors. Preisich and Schultz[11] found tubercle bacilli under the nails of 14 out of 66 children examined. The possibility of infection being spread by ordinary coughing is considered by Williams[12] to be negligible, as hospital nurses and doctors so seldom acquire phthisis, though frequently exposed to it in this manner. Newsholme,[13] while considering that compulsory notification is bound to come, deprecates any undue haste in pressing for it. He advocates voluntary notification, and points out how a medical officer of health can assist and support the general practitioner in enforcing treatment and prophylaxis. He advocates thorough disinfection of the houses of notified cases, and careful inquiry as to the source of each case to check continued exposure of the patient and others. Alcohol and want of domestic or occupational hygiene are specially important points. He thinks medical officers of health should examine sputum gratuitously for medical men. Billings,[14] however, says that sputum examination in New York does not lead to notification ; out of 8,500 examinations, only 3,200 are reported by physicians. Lawson quotes Larnier's opinion that compulsory notification would necessitate an enormous expenditure on asylums and sanatoria, but disagrees with his conclusion that it is therefore undesirable. In Norway, he states, notification is limited to expectorating cases. Behring[15] advocates the addition of formalin to milk in the proportion of 1 in 25,000 as a preventive measure. Tunnicliffe and Rosenheim have shown that formic aldehyde up to 1 in 5,000 in milk has no ill effect on healthy children.

[1] Med. Rec., Jan. 9, 1904, p. 55. [2] Pract., Nov. 1903, p. 666. [3] Ibid., p. 694. [4] Sem. Med., Dec. 2, 1903, p. 392. [5] Scot. Med. and Surg. Jour., Feb. 1904, p. 145. [6] Sem. Med., Dec. 23, 1903, p. 410. [7] Med. Rec., Jan. 2, 1904, p. 61. [8] Amer. Jour. Med. Sci., Oct. 1903, p. 674. [9] Loc. cit., p. 147. [10] Med. Rec., Dec. 19, 1903, p. 974. [11] Scot. Med. and Surg. Jour., Feb. 1904, p. 145. [12] Lancet, Jan. 30, 1904, p. 278. [13] Ibid., p. 282. [14] Med. Rec., Dec. 12, 1904, p. 959. [15] Therapist, Feb. 15, 1904, p. 18.

